# Flow cytometric identification and cell-line establishment of macrophages in naked mole-rats

**DOI:** 10.1038/s41598-019-54442-1

**Published:** 2019-11-29

**Authors:** Haruka Wada, Yuhei Shibata, Yurika Abe, Ryo Otsuka, Nanami Eguchi, Yoshimi Kawamura, Kaori Oka, Muhammad Baghdadi, Tatsuya Atsumi, Kyoko Miura, Ken-ichiro Seino

**Affiliations:** 1 Division of Immunobiology, Institute for Genetic Medicine, Hokkaido University, Sapporo, Japan; 2 Department of Rheumatology, Endocrinology and Nephrology, Graduate School of Medicine and Faculty of Medicine, Hokkaido University, Sapporo, Japan; 3 Department of Aging and Longevity Research, Faculty of Life Sciences, Kumamoto University, Kumamoto, Japan; 4 Biomedical Animal Research Laboratory, Institute for Genetic Medicine, Hokkaido University, Sapporo, Japan; 5 Center for Metabolic Regulation of Healthy Aging, Faculty of Life Sciences, Kumamoto University, Kumamoto, Japan

**Keywords:** Peritoneal macrophages, Phagocytes

## Abstract

Naked mole rats (NMRs) have extraordinarily long lifespans and anti-tumorigenic capability. Recent studies of humans and mice have shown that many age-related diseases, including cancer, are strongly correlated with immunity, and macrophages play particularly important roles in immune regulation. Therefore, NMR macrophages may contribute to their unique phenotypes. However, studies of the roles of macrophages are limited by material restrictions and the lack of an established experimental strategy. In this study, we developed a flow cytometric strategy to identify NMR macrophages. The NMR macrophages were extractable using an off-the-shelf anti-CD11b antibody, M1/70, and forward/side scatter data obtained by flow cytometry. NMR macrophages proliferated in response to human/mouse recombinant M-CSF and engulfed *Escherichia coli* particles. Interestingly, the majority of NMR macrophages exhibited co-staining with an anti-NK1.1 antibody, PK136. NK1.1 antigen crosslinking with PK136 results in mouse NK cell stimulation; similarly, NMR macrophages proliferated in response to NK1.1 antibody treatment. Furthermore, we successfully established an NMR macrophage cell line, NPM1, by transduction of Simian virus 40 early region that proliferated indefinitely without cytokines and retained its phagocytotic capacity. The NPM1 would contribute to further studies on the immunity of NMRs.

## Introduction

Naked mole rats (NMR, *Heterocephalus glaber*) are subterranean rodents found in the eastern Horn of Africa that breed in eusocial colonies of up to about 300 individuals with only one breeding female and a few breeding males^1^. Although their sizes (average body weight: 35 g) are almost the same as those of house mice (*Mus musculus*), they have a much longer life span (up to 32 years) than expected based on body size, making them the longest-lived rodent species in the world. In addition to their longevity, they spend at least about 80% of their lives without a loss of vital activity, reproductive ability, and cardiovascular function. Furthermore, they do not show an age-associated acceleration in mortality risk. Furthermore, spontaneous oncogenesis, associated with age, is extremely rare in NMRs^2–5^.

NMRs have been the focus of recent research as new model animals to study tumour resistance^6^. Several mechanisms underlying cancer resistance in NMRs have been also reported, including hypersensitivity to contact inhibition (early contact inhibition)^7^, secretion of high-molecular-mass hyaluronan^8^, triggering apoptosis or senescence when either p53 or Rb tumour suppressors is inactivated^7,9^, high levels of p53^10^, and overregulation of telomerase activity^11^. High fidelity protein synthesis^12–14^, more active antioxidant pathway^13,15^ more efficient DNA double-strand break repair^16^, and high levels of autophagy^17^ may also contribute to the tumour resistance of NMRs. Interestingly, the anti-tumorigenic feature remains in induced pluripotent stem cells (iPSCs) derived from NMRs via Arf regulation and the disruption of Eras^9^. Innate and acquired immune systems are also related to tumour progression in many animal species^18^. However, the contribution of the immune system to anti-tumorigenicity in NMR is still unknown. Cheng *et al*. first reported about the features of NMR macrophages compared to ICR mice; higher phagocytotic activity, less apoptotic to Toll-like receptor stimulation and higher expression level of NF-κB protein^19^. But the information about NMR immune cells is still limited, and methodologies for investigating immune cells in the species have not been established.

Macrophages play important roles in both innate and adaptive immunity and contribute to tumorigenesis/anti-tumorigenesis^20^ and aging-related diseases, such as atherosclerosis^21^. As mentioned above, NMR exhibits extraordinary resistance to malignant neoplasms and cardiovascular aberrances. Therefore, it is possible that NMR macrophages have special functions or features, distinct from macrophages of other species. Although it has already shown an existence of NMR cells with phagocytic activity and a feature of their cytokine production^19^, precise protocol for identifying the NMR phagocytes has not been indicated, such as a method using flow cytometry. In this study, we first established a strategy for the identification of NMR macrophages using off-the-shelf antibodies and flow cytometry. Then, we established an NMR macrophage cell line based on the flow cytometry information. Breeding methods for NMRs have been established but require special facilities and are less feasible than mouse and rat breeding methods. Therefore, sacrificing NMRs to obtain immune cells for every experiment is not always reasonable. Cell lines are a strong tool for understanding cell functions and have been important for immunology research^22,23^. We successfully established an NMR macrophage cell line that proliferated without cytokines. The cells shared features with primarily isolated NMR macrophages. The unique features of NMR macrophages were identified in this study and the novel macrophage cell line would contribute to further studies of the curious aspects of NMR immune systems.

## Results

### Identification of NMR macrophages in the spleen, bone marrow, and peritoneal cavity

**Figure 1:**
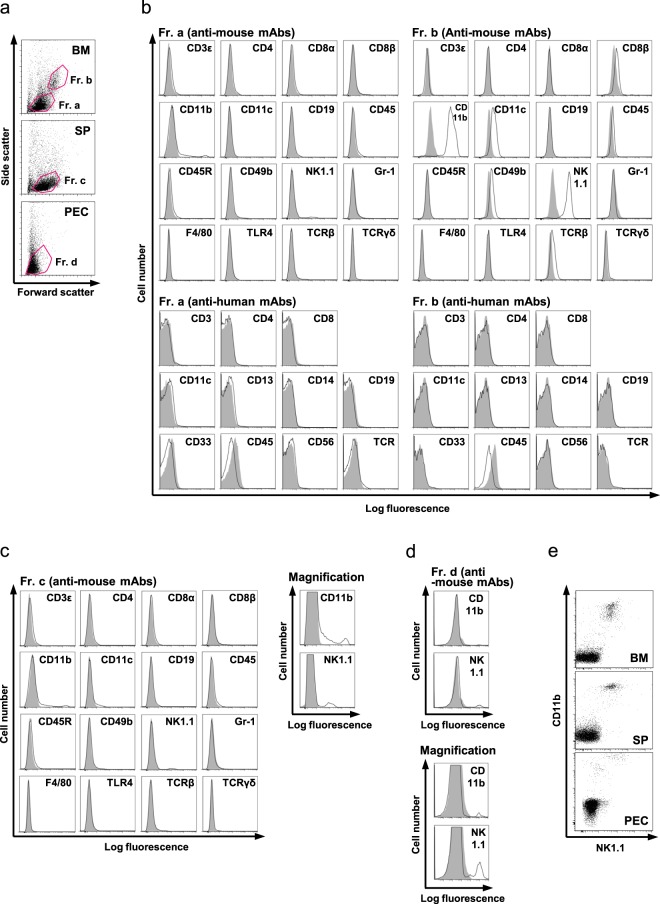
Flow cytometric analysis of NMR cells. (**a**) Freshly isolated NMR bone marrow cells (BM), spleen cells (SP), and peritoneal cavity cells (PEC) were analysed by flow cytometry. Forward scatter and side scatter profiles for each cell type are shown. (**b–d**) BM (**b**), SP (**c**), or PEC (**d**) cells were stained with anti-mouse or anti-human monoclonal antibodies and analysed by flow cytometry. Cells in each fraction (Fr.) are shown. Open histograms show specific antibody staining, and grey histograms show each isotype control. Enlarged images of anti-CD11b (M1/70) and anti-NK1.1 (PK136) were shown in the right side (SP) and the underside (PEC). (E) BM, SP, and PEC cells were co-stained with anti-mouse/human CD11b (M1/70) and anti-NK1.1 (PK136) antibodies and analysed by flow cytometry.

The features of NMR immune cells by flow cytometry and effective antibodies for their identification have not been established. We obtained cells from the bone marrow, spleen, and peritoneal cavity (PEC) for flow cytometry and used commercially available anti-mouse/ anti-human/anti-rat antibodies to detect NMR immune cells (Figs. 1a–e and S1). As reported previously^19^, NMR spleens exhibited a sigmoidal shape and a slender form compared to mouse spleens (Fig. S2). Flow cytometry revealed that there are two different bone marrow cell populations that can be distinguished by size (fraction (Fr.) a and b) and a single population in each of spleen cells and PECs (Fr. c and d, respectively) (Fig. 1a). We found that an anti-mouse/human CD11b antibody (clone: M1/70), also known as Mac-1, reacted to large cells (Fr. b) in the bone marrow, spleen, and PEC (Fig. 1b–d). CD11b is a marker of mouse and human macrophages. This antibody has cross reactivity to both human and mouse macrophages^24^. CD11b proteins have relatively high amino acid sequence homology among humans, mice, and NMRs (NMR vs. mouse: 72.77%, NMR vs. human: 75.35%, mouse vs. human: 74.59%) (Fig. S3); therefore, the epitope that binds to the M1/70 antibody may be shared among them. Further, we found that the anti-mouse NK1.1 antibody (clone: PK136) also reacted against bone marrow cells in Fr. b, spleen cells, and PEC (Fig. 1b–d). NK1.1, also known as CD161 or killer cell lectin-like receptor subfamily B member 1C (Klrb1c), is a marker of mouse NK cells^25^ but only in certain strains, including C57BL/6, FVB/N, and NZB^26^. Interestingly, most CD11b-positive cells co-expressed NK1.1 (Fig. 1e). Thus, we first identified useful off-the-shelf antibodies, anti-mouse/human CD11b antibody (clone: M1/70) and anti-mouse NK1.1 antibody (clone: PK136), for analyses of NMR immune cells. In these experiments, we could not find any anti-human antibody examined with reactivity against NMR cells (Fig. 1b). Anti-rat antibody against CD68, a macrophage marker, did not react with any NMR cells (Fig. S1).

### Bone marrow, spleen, and PEC cells proliferated in response to mouse/human M-CSF

**Figure 2:**
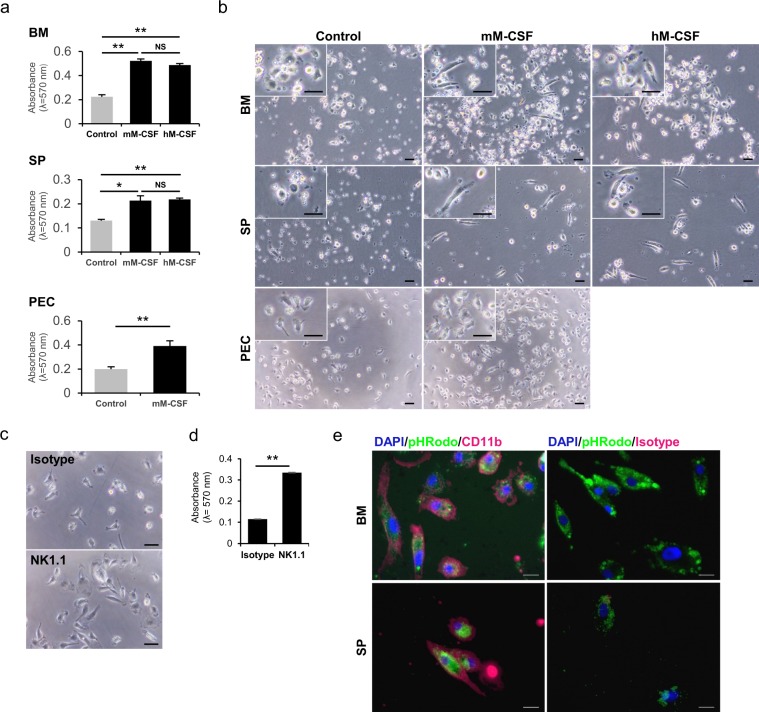
NMR BM, SP, and PEC contained cells with macrophage features. (**a,b**) Fleshly isolated NMR BM, SP, and PEC cells were cultured with 20 ng/ml mouse or human macrophage-colony stimulating factor (mM-CSF or hM-MCSF, respectively) or saline (control). (**a**) After 7 days, cell proliferation was assessed by MTT assays. Representative results are presented as means ± SD. NS: not significant. *p < 0.05, **p < 0.01, as determined by Student’s *t*-tests. (**b**) Phase contrast images of resulting cells on day 7. High magnification images are shown in the upper-left. Scale bar: 20 μm (**c,d**) PEC cells were cultured on 5 μg/ml anti-NK1.1 antibody- or corresponding isotype control antibody-coated plates for 7 days. (**c**) Phase contrast images of resulting cells are shown. Scale bar: 20 μm (**d**) Cell proliferation was assessed by MTT assays. Representative results are presented as means ± SD. **p < 0.01, Student’s *t*-test. (**e**) Phagocytotic activity analysis. BM and SP cells were cultured for 8 days with 20 μg/ml mM-CSF, pHrodo-labeled *E. coli* particles were added, and cells were incubated for 2 hours. Cells were observed by fluorescent microscopy after fixation and anti-CD11b antibody or corresponding isotype control immunostaining (red). Nuclei were stained by DAPI (blue). Merged fluorescent images are shown. Scale bar: 20 μm. Only phagocytosed pHrodo-labeled *E. coli* particles show green fluorescence (green).

We found that the NMR bone marrow, spleen, and PEC samples include CD11b-positive cells. We further evaluated whether the cells proliferate in response to mouse/human macrophage colony stimulating factor (M-CSF). As shown in Fig. 2a, 20 μg/ml mouse/human M-CSF induced significant proliferation of NMR bone marrow, spleen, and PEC cells. The addition of human/mouse M-CSF to the culture resulted in macrophage-like elongated adherent cells (Fig. 2b). There were no significant differences between the effects of mouse and human M-CSF (Fig. 2a). Both M-CSF and its receptor CSF1R had relatively high (about 70–80%) amino acid sequence homology among species (Fig. S4a,b), and this may explain the cross-reactivity of mouse/human M-CSF in NMR cells.

### NK1.1 antibody stimulation induced NMR cell activation

NK1.1 recognises Klrb1c or Nkrp1c in mice^26^. Klrb1c is an NK cell-activating receptor, and cross-linking using an NK1.1 antibody result in NK cell activation, including cell proliferation^27^. We showed that the majority of CD11b-positive cells in NMR co-express NK1.1 (Fig. 1e). This observation motivated us to evaluate whether stimulation by an NK1.1 antibody induces the activation of NMR cells, as observed in mouse cells. Freshly isolated NMR PECs including CD11b-positive NK1.1-positive cells were cultured on NK1.1 antibody- or isotype control antibody-coated plates for 1 week. A morphological analysis revealed that NK1.1 stimulation resulted in large-sized cells with extended pseudopods compared to the control cells (Fig. 2c). Further, we also observed significant cell proliferation in response to NK1.1 stimulation (Fig. 2d). Similar tendencies were also observed for NMR bone marrow cells and splenocytes (data not shown). These results suggested that NMR cells are activated in response to NK1.1 stimulation.

### Phagocytotic activity of NMR cells

Phagocytotic activity is an important characteristic of macrophages. Therefore, we analysed the phagocytotic function of cells using the pHrodo system (Fig. 2e). In this system, only engulfed *Escherichia coli* particles emit green fluorescence by a reduction in pH in phagosomes. We cultured bone marrow cells or splenocytes with mouse M-CSF for 8 days and analysed phagocytotic activity. Immunofluorescent staining showed that almost 100% of the resulting adherent cells induced by M-CSF were positive for CD11b (Fig. 2e). Further, the CD11b^+^ cells exhibited green fluorescence, indicating that they engulfed *Escherichia coli* particles. Importantly, phagocytotic activity was not observed at 4 °C, conditions in which cell function would be reduced (data not shown). These results indicated that the NMR cells in the bone marrow and spleen in response to M-CSF had phagocytotic activity. Thus, cells with macrophage features reside, at minimum, in the bone marrow, spleen, and peritoneal cavity in NMRs.

### Identification of macrophages in NMR

**Figure 3:**
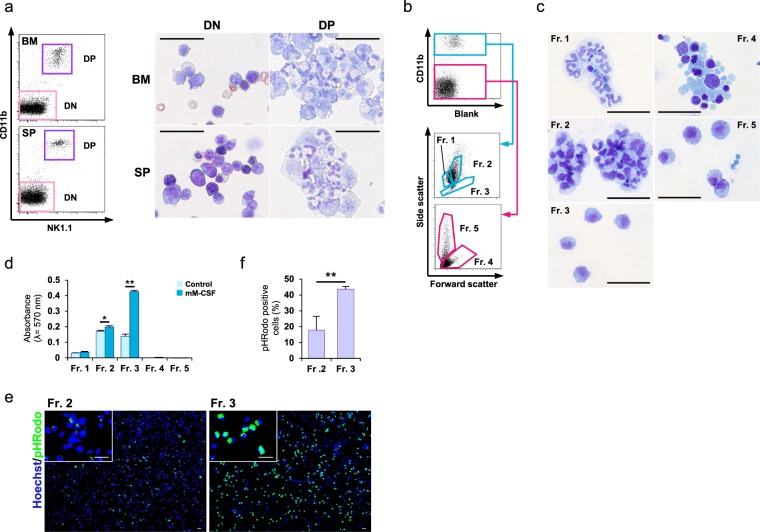
Flow cytometric detection of NMR macrophages. (**a**) CD11b and NK1.1 double-positive (DP) and double-negative (DN) cells were sorted using a cell sorter, and smears were prepared using Cytospin. Diff-Quik-stained cells are shown. Scale bar: 20 μm (**b**) Gating strategy for NMR BM cells to detect macrophages. CD11b-positive (blue square) or -negative (red square) cells were further subdivided into fractions depending on forward/side scatter appearance. (**c**) Microscopic features of each fraction. Sorted cells were stained by Diff-Quik. Scale bar: 20 μm (**d**) Cell fractions depending on the strategy shown in (**b**) were cultured with mM-CSF or saline (control) for 7 days. Cell proliferation was assessed by MTT assays. Representative results are presented as means ± SD. *p < 0.05, **p < 0.01, Student’s *t*-test. (**e**) Phagocytotic activity of freshly isolated Fr. 2 or 3 cells was analysed. pHrodo-labeled *E. coli* particles were added to the culture and incubated for 2 hours. Phagocytosed *E. coli* particles were detected by green fluorescence (green). Nuclei were stained by Hoechst 33342 (blue). Scale bar: 20 μm. (**f**) Counts of green fluorescence-positive cells for Fr. 2 and 3 were analysed. Representative results are presented as means ± SD. **p < 0.01, Student’s *t*-test.

In a cytological analysis of NMR CD11b^+^ cells, bone marrow and spleen CD11b and NK1.1 double-positive cells contained some stab-nuclear cells and cells with large cytoplasmic surfaces and vacuoles compared to double-negative cells (Fig. 3a). These results indicated that the NMR CD11b/NK1.1 double-positive cells include various types of cells. CD11b is also a surface marker of neutrophils. Since there are only two available antibodies for NMR immune cell discrimination, further strategies for macrophage identification, in addition to the use of an anti-CD11b or anti-NK1.1 antibodies are needed. We focused on forward scatter (FSC) and side scatter (SSC) analyses by flow cytometry for the precise identification of macrophages in NMR. CD11b-positive and -negative cells were subdivided by FSC and SSC (Fig. 3b), and sorted cells were observed by Giemsa staining and optical microscopy (Fig. 3c). In the CD11b-positive population, cells in Fr. 1 were ~8 μm with stab/segmented-nuclei, similar to neutrophils. Cells in Fr. 2 resembled Fr. 1 cells, but they were slightly larger (~10 μm) and had many small vacuoles. Cells in Fr. 3 were ~12 μm and had large cytoplasmic areas. They also had many vacuoles, like Fr. 2 cells, but the nuclei and general appearance were quite different; the nuclei were poorly stained and cells were not stab/segmented. Importantly, they uniquely had pseudopodia, unlike the cells in other fractions. In the CD11b-negative population, we observed cells of various sizes, indicating that Fr. 4 contained many kinds of cells. Fr. 5 cells had vacuoles and granules. In general, neutrophils show higher FSC and SSC compared to those of monocytes/macrophages. Based on the morphological analysis and flow cytometry profiles, CD11b^+^ Fr. 3 cells were likely macrophages.

To further identify macrophages, we evaluated M-CSF reactivity in each fractionated cell. As shown in Fig. 3d, Fr. 3 cells proliferated efficiently in response to M-CSF. Using the pHrodo system, phagocytotic activity was observed in Fr. 2 and 3 cells but was more efficient in Fr. 3 cells than in Fr. 2 cells (Fig. 3e,f). Neutrophils and macrophages both express the marker CD11b and have phagocytotic activity. Combined with the poor reactivity to M-CSF, Fr. 2 cells were likely neutrophils rather than macrophages.

We further evaluated the fractionalized cells using qPCR (Fig. S5a). Fr.3 cells expressed MHC class 2 and CD86 at a relatively higher level than other fractionalized cells. This result implied that Fr.3 cells were antigen-presenting cells similar to macrophages or dendritic cells. Therefore, we attempted to distinguish them by additionally analyzing the expression of marker genes *Itgax* (CD11c), a well-known dendritic cell marker. However, there were no significant differences between the expression levels in the cells of all fractions (Fig. S5a). Flow cytometry analysis also yielded similar results (Fig. S5b). In contrast, some macrophage marker genes such as *Emr1* (F4/80), *CD68*, and *Siglec1* (CD169) were highly expressed in Fr. 3 cells compared with other fractionalized cells (Fig. S5a). These results support the notion that it was appropriate to define Fr. 3 cells as macrophages rather than dendritic cells. We further examined the expression of M2 macrophage marker genes, *Mrc1* (CD206) or *CD163*, and also of innate immunity-related genes such as *Tlr3*, *Tlr4*, *Ifnb*, or *Tnfa*, but no characteristic expression to the Fr. 3 was identified (Fig. S5a).

### Establishment of an NMR macrophage cell line

**Figure 4:**
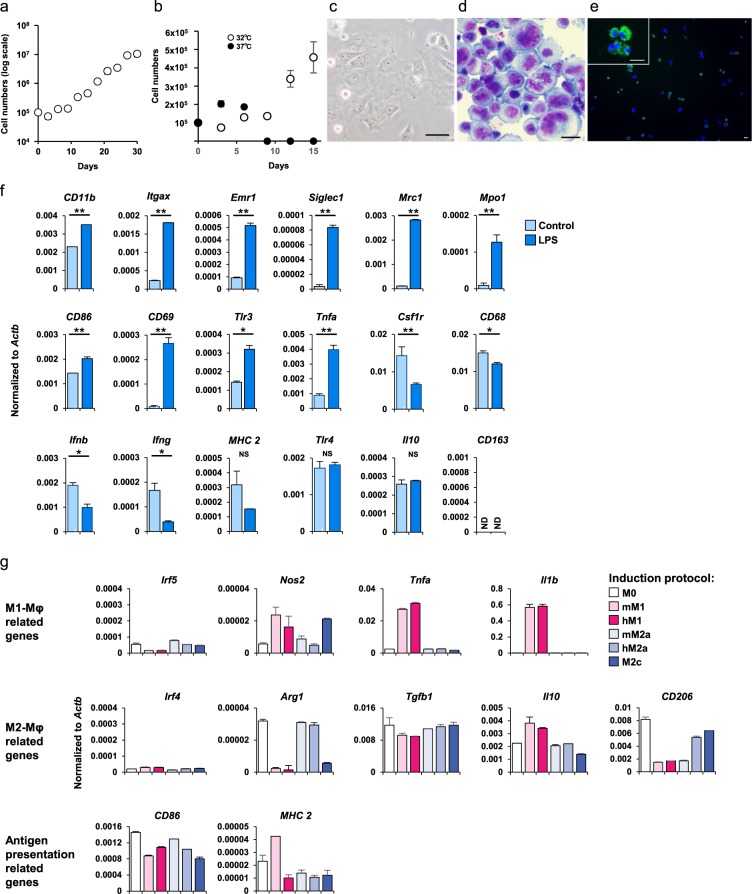
Features of NPM1 cells. (**a**) Growth curve for NPM1. Cells were passaged every 3 days and counted. Cell counts are presented as means ± SD. (**b**) NPM1 cells were cultured at 32 °C (open circles) or 37 °C (closed circles). Cell counts are presented as means ± SD. (**c**) Phase contrast image of NPM1. Scale bar: 50 μm (**d**) NPM1 was stained by Diff-Quik. Scale bar: 20 μm (**e**) The phagocytotic activity of NPM1 was analysed. pHrodo-labelled *E. coli* particles were added to the culture and incubated for 2 hours. Phagocytosed *E. coli* particles were detected by green fluorescence (green). Nuclei were stained by Hoechst 33342 (blue). Scale bar: 20 μm (f) NPM1 was cultured with 1 μg/ml LPS or saline (control). After 24 hours, gene expression was analysed by quantitative-PCR. Gene expression levels are presented as means ± SD. NS: not significant. ND: not detected. *p < 0.05, **p < 0.01, Student’s *t*-test. (**g**) NPM1 cells were cultured after treatment with several cytokines and reagents. After 24 hours, gene expression was analysed using quantitative-PCR. Gene expression levels are presented as the mean ± SD. Representative results are shown. M0: control, mM1: 10 μg/ml LPS + 25 ng/ml mouse IFN-γ, hM1: 10 μg/ml LPS + 25 ng/ml human IFN-γ, mM2a: 25 ng/ml mouse IL-4, hM2a: 25 ng/ml human IL-4, M2c: 1 μM dexamethasone.

NMRs are not commonly used as live animals for experiments owing to unique rearing conditions. Accordingly, cell lines would facilitate biological studies of NMRs. Therefore, we established an NMR macrophage cell line. In mice and humans, there are several well-known macrophage cell lines. For the establishment of an immortalized macrophage line, we used simian virus 40 early region (SV40ER) transfection, a conventional method^28^. SV40ER produces two oncoproteins; large T antigen (SV40-LT) and small T antigen (SV40-ST). SV40-LT binds both p53 and Rb, important tumour suppressor genes and inhibits their functions. SV40-ST inhibits the activity of the protein phosphatase 2A^29^. We prepared PEC-macrophages by culturing freshly isolated PECs with mouse M-CSF for more than 7 days. Then, the macrophages were transfected with SV40ER, and we successfully obtained a cell line (NPM1; Naked mole rat peritoneal cavity macrophage-1). NPM1 proliferated slowly but continuously without any cytokines (Fig. 4a), whereas freshly isolated PEC cells with M-CSF stimulation ceased their growth before 5th passage. According to the NMR’s body temperature, we cultured NPM1 at 32 °C and they proliferated for more than 30 passages (data not shown). Unlike the infinite growth at 32 °C, NPM1 showed growth arrest at 37 °C (Fig. 4b), indicating that the cell line retained thermal susceptibility. NPM1 cells had a large cytoplasm and nucleus compared to those of primary NMR PECs (Fig. 4c,d). NPM1 exhibited a similar phagocytotic capacity to that of freshly isolated macrophages (Fig. 4e). These findings suggested that NPM1 had indefinitely proliferative capacity as well as phagocytic functions. Results of flow cytometry analysis revealed that NPM1 cells express molecules which react with anti-mouse/human/rat CD11b, anti-mouse CD11c, and anti-mouse NK1.1, but not with anti-rat CD68, antibodies (Fig. S6a,b). Further, we analysed gene expression changes in NPM1 cells in response to LPS stimulation (Fig. 4f). At 24 hours after stimulation, *CD11b*, *Itgax, Emr1, Siglec1, Mrc1, Mpo1, CD86, CD69*, *Tlr3*, and *Tnfa* expression levels increased, but *Csf1r, CD68, Ifnb*, and *Ifng* were decreased, further, *MHC class 2, Tlr4, IL-10* and *CD163* expression did not change, almost consistent with a previous finding that used M-CSF cultured macrophages^19^. Moreover, we assessed the features of NPM1 cells under the condition of induced polarization. Based on a previous study^30^, we exposed NPM1 cells to several mouse/human cytokines and reagents and evaluated their M1/M2 polarizations according to their gene expression (Fig. 4g). In response to M1-inducible conditions, increased expression of M1-related genes were observed compared with that under the M0 condition. Contrastingly, under M2-inducible conditions, the NPM1 cells showed no change in M2-related gene expression compared with that under the M0 condition. Interestingly, the expression of *Arg1* and *CD206*, M2-related genes, remained unchanged under M2-inducible conditions but markedly decreased under M1-inducible conditions. These results suggested that the NPM1 cells were favorable toward M2 in the steady state.

## Discussion

NMRs are an exceptional animal with extremely high tumour resistance and a much longer life span than expected based on their body weight. Recent biomedical research has revealed a deep involvement of the immune system in longevity. Macrophages have critical roles in the control of age-related diseases, such as atherosclerosis and tumorigenesis. Accordingly, investigations of NMR macrophages would improve our understanding of its biomedical features. However, precise protocol for identifying the NMR macrophages has not been established, such as a method using flow cytometry as we have shown in this study.

Monoclonal antibodies are very important and useful to push immunology research forward in the era of molecular biology. We screened off-the-shelf anti-mouse/human monoclonal antibodies for the identification of NMR macrophages. We found that an anti-mouse/human CD11b monoclonal antibody (clone M1/70) could react with NMR macrophages. M1/70 recognises CD11b, also known as Mac-1 antigen, integrin alpha M (*Itgam*), a protein subunit that forms a heterodimeric integrin alpha-M beta-2 (*αMβ2*) molecule^31,32^. M1/70 is a commonly used monoclonal antibody to identify myeloid cells, including macrophages and monocytes. It has cross-reactivity against human, mouse, and rabbit CD11b^24^. In this study, we found that there is a M1/70 reactive population in spleen, bone marrow, and PEC of NMR (Fig. 1). However, owing to a potential lack of specificity, CD11b staining alone is insufficient to identify macrophages. Therefore, we developed an NMR macrophage identification strategy using FSC/SSC analysis by flow cytometry in combination with CD11b staining; we confirmed the accuracy of the method by evaluating M-CSF reactivity and phagocytotic activity. Detailed analysis using qPCR revealed that Fr. 3 cells expressed *MHC class 2* and *CD86* at higher levels than other fraction cells. This observation indicates that Fr. 3 cells are antigen-presenting cells similar to macrophages and dendritic cells. Furthermore, dendritic cells are also known to express CD11b similar to macrophages^33^. Therefore, we performed another analysis to identify whether Fr. 3 cells were macrophages or dendritic cells. The expression of *Itgax* (CD11c), a well-known dendritic cell marker, but known to also express in some kinds of macrophages^34^ was detected in Fr. 3 cells at mRNA (Fig. S5a) and protein levels (Fig. S5b), but the expression level was not markedly high compared to other fraction cells. As indicated by the meaning of the term “dendrites”, dendritic cells are conventionally identified by their characteristic morphology of “dendrites”^35^ but the Fr. 3 cells did not exhibit a dendritic structure (Fig. 3c). Therefore, it is unreasonable to consider them as dendritic cells. Moreover, dendritic cells are known to exhibit phagocytic activity, although poorer than that exhibited by macrophages^36^. As shown in Fig. 3e, Fr. 3 cells showed efficient phagocytic activity. Moreover, dendritic cells are known to express lower level of CSF1R than macrophages^37^, which leads to poorer response. As shown in Fig. 3d, Fr. 3 cells efficiently proliferated in response to M-CSF. Considering these observations along with the morphological features (Fig. 3c), phagocytic activity (Fig. 3e,f), effective cell proliferation in response to M-CSF stimulation (Fig. 3d), and the gene expression profiles (Fig. S5a), we finally concluded that the characteristics exhibited by NMR bone marrow cells in Fr. 3 could define them as macrophages.

The anti-NK1.1 antibody (clone PK136) was also found to bind to NMR cells as well as the anti-CD11b antibody. PK136 recognises NK1.1 antigen, also known as CD161, killer cell lectin-like receptor subfamily B member 1C (Klrb1c) or Nkrp1c, an NK cell-activating receptor in mice. In humans, lectin-like transcript 1 (LLT1) is the functional ligand for CD161^26^. Although NK1.1 is commonly used as an NK cell marker in mouse immunology research, its ligand in mice has not been identified^38^. Klrb1c is an NK cell activation receptor; its cross-linking with PK136 results in NK cell activation in mice^27^. Interestingly, most NMR CD11b-positive macrophages also expressed NK1.1. CD11b/NK1.1 double-positive cells in NMR showed the morphological features of myeloid cells, macrophages, and granulocytes, rather than lymphocytes. Further, they showed phagocytotic activity and an M-CSF response. Therefore, we concluded that they are macrophages, expressing a molecule which reacts with PK136.

Recent research has shown that the danger signal monosodium urate (MSU) crystals induces NK1.1 expression in macrophages and alters their function^39^. However, steady-state macrophages do not generally express NK1.1 in mice. Therefore, it was speculated that NMR macrophages that express the NK1.1 antigen in the steady state function not only as macrophages but also as NK cells. To the best of our knowledge, this is the first study to indicate that macrophages in naïve state share the physiological characteristics of NK cells; this might explain the extraordinary immune function of NMRs. An analysis showed that NMR macrophages cultured on a PK136-coated plate proliferated extensively. Therefore, the surface molecule identified by the anti-NK1.1 antibody in NMR was expected to function in NMR immunity. We tried to identify the antigen recognised by PK136, i.e., the ortholog of Klrb1c in NMR, by a genome database analysis, but so far we have been unsuccessful (data not shown). The identification of the antigen recognised by the anti-NK1.1 antibody in NMR would improve our understanding of NMR macrophages.

Despite the interesting features of NMR macrophages, availability of the materials for research is limited. To improve accessibility, we established an NMR macrophage cell line by the enforced expression of SV40ER. The cell line, named NPM1, as far as we observed, it proliferated indefinitely at 32 °C. Interestingly, as previously reported in the case of NMR primary skin fibroblasts^40^, NPM1 did not survive at 37 °C probably due to the low body temperature (around 32 °C) of NMRs. These results indicate that the temperature susceptibility of NPM1 at 37 °C is probably independent on p53 and Rb pathway because SV40 Large T inhibits their activity. Thus, the newly developed NMR macrophage cell line NPM1 would contribute not only to immunological research but also to cellular biological research of NMRs such as temperature susceptibility.

NMRs have a smaller thymus and a larger spleen red/white pulp ratio than those of mice (data not shown). These differences suggest that NMRs have a higher myeloid cell and lower lymphoid cell frequencies compared to those in mice, i.e., macrophages are particularly abundant in NMRs. Further, NMR macrophages, which share NK cell marker expression even in the steady state, may have unexpected functions compared to conventional macrophages. It has been reported that clearance of senescent cells by inducing cell death in these cells extends the life span of mice^41^. NK cells kill abnormal cells, including senescent cells^42^. Therefore, we tried to detect killing activity of NMR macrophage using freshly isolated macrophage and NPM1. Unfortunately, we could not find killing activity against SV40ER transfected NMR fibroblasts, YAC-1 and K562 cells as far as we tested (data not shown). Nonetheless, the extraordinarily long life span and anti-tumorigenic features of NMRs suggest that the species has a mechanism by which macrophages kill senescent cells by an NK cell-like function, followed by the efficient clearance of dead cells by the macrophage function. Thus, further studies of NMR macrophages may reveal their roles in the unique long lifespans and anti-tumorigenesis activity in the species.

## Methods

### Animals

The Ethics Committees of Hokkaido University (Approval No. 17-0112) approved of all procedures, which were in accordance with the Guide for the Care and Use of Laboratory Animals (National Institutes of Health, Bethesda, MD, USA). The NMR colonies are maintained at the Biomedical Animal Research Laboratory, Institute for Genetic Medicine, Hokkaido University/Laboratory for Molecular Biology of Aging and Longevity, Faculty of Life Sciences, Kumamoto University. NMRs were obtained from a single colony.

### Isolation of NMR bone marrow cells and splenocytes

NMRs were euthanised by isoflurane anesthetisation followed by cervical dislocation. The limbs and spleens were used for cell preparation. Muscles and cartilage tissue were removed from the extremities, and the bone marrow cavity was rinsed using a blunt needle and syringe with PBS. Cells were processed into a single cell suspension without red blood cell lysis. Spleens were homogenised by the frosted ends of slide glasses. Cells were collected; after centrifugation, red blood cells were lysed using a hypo-osmotic solution.

### Collection of NMR peritoneal cavity cells (PECs)

PECs were collected according to methods described in previous reports^43,44^. Five days before collecting PECs, 1 ml of 3% thioglycolate was injected intraperitoneally. On the day of collection, NMRs were anesthetised by the intraperitoneal injection of a cocktail of anaesthetics, including medetomidine hydrochloride (Nippon Zenyaku Kogyo Co., Ltd., Tokyo, Japan), midazolam (Teva Takeda Pharma Ltd., Nagoya, Japan), butorphanol (Meiji Seika Pharma Co., Tokyo, Japan)^45^, and isoflurane inhalation. After sterilising NMR abdomens with 70% (v/v) ethanol, 5–10 ml of heparin-supplemented physiological saline (final heparin concentration: 5 U/ml) was intraperitoneally injected. To avoid frequent puncture and to perform injection and collection simultaneously, a 23 G butterfly needle was used. After injection, the abdomen was gently massaged and natural drips were collected from the butterfly needle. These procedures were repeated 5 to 10 times. After collecting PECs, NMRs remained in good condition. Contaminated red blood cells were lysed by lysis buffer followed by centrifugation of the fluid.

### Cell culture

NMR cells were cultured in RPMI1640 medium (Wako, Osaka, Japan) supplemented with l-glutamate, 10% (vol/vol) foetal bovine serum (FBS) (EQUITECH-BIO, INC., Kerrville, TX, USA or SIGMA-ALDRICH, St. Louis, MO, USA), 1% penicillin/streptomycin (Nacalai Tesque, Kyoto, Japan), 0.1 mM non-essential amino acids (Nacalai Tesque), and 0.1 mM 2-2-mercaptoethanol. NMR cells were incubated at 32 °C in a 5% O_2_ and 5% CO_2_ atmosphere.

### Flow cytometry and antibodies

All sample cells were suspended in PBS supplemented with 0.5% (vol/vol) FBS and 2.5 mM EDTA. Sample cells were preincubated in 5% rat and mouse serum at room temperature for 5 minutes. Flow cytometry was performed using the FC500 instrument (BeckmanCoulter, Brea, CA, USA), FACS Canto II, or FACS Aria II (BD Biosciences, Franklin Lakes, NJ, USA) and data were analysed using FlowJo software (TOMY DIGITAL BIOLOGY CO., LTD., Tokyo, Japan). The following fluorescence-conjugated monoclonal antibodies were used in this study: anti-mouse CD3e (145-2C11), anti-CD4 (GK1.5), anti-CD8α (53-6.7), anti-CD8β (YTS156.7.7), anti-CD11b (M1/70), anti-CD11c (N418), anti-CD19 (1D3), anti-CD45 (30-F11), anti-CD45R (RA3-6B2), anti-CD49b (Ha1/29), anti-NK-1.1 (PK136), anti-Gr-1 (RB6-8C5), anti-F4/80 (BM8), anti-TCRβ (H57-597), anti-TCRγδ (eBioGL3), anti-human CD3 (UCHT1), anti-CD4 (OKT4), anti-CD8 (OKT-8), anti-CD11b (M1/70), anti-CD11c (3.9), anti-CD13 (WM15), anti-CD14 (HCD14), anti-CD19 (HIB19), anti-CD45 (HI30), anti-CD56 (MEM-188), anti-CD33 (WM53), and anti-TCR (IP26). These antibodies and corresponding isotype controls were purchased from Biolegend (San Diego, CA, USA), eBioscience (San Diego, CA, USA), BD Pharmingen (San Jose, CA, USA), BD Biosciences, and Biological Laboratories (Campbell, CA, USA). Anti-Rat CD68 (REA237) antibody and its isotype control antibody (REA293) were purchased from Miltenyi Biotec (San Diego, CA, USA). For analysis, live cells were gated based on forward and side scatter as well as a lack of 4′,6-diamidino-2-phenylindole (DAPI) or propidium iodide (PI) uptake.

### Cross-linking stimulation by an anti-NK1.1 antibody

A 96-well flat bottom plate was coated with 5 μg/ml purified anti-mouse NK-1.1 antibody (BioLegend) or 5 μg/ml purified Mouse IgG1, κ, Isotype Control Antibody (BioLegend) incubated for 1 day. The supernatant was then removed, and cells were cultured for a week.

### MTT assay

Cell proliferation was assessed by MTT assays using the Cell Proliferation Kit I (Roche, Basel, Switzerland) after 7 days of culture. Formazan quantification was performed using Multiscan FC (Thermo Fisher Scientific, Waltham, MA, USA) at a wavelength of 570 nm.

### Phagocytosis assay

NMR cells were used after 8 days of culture with 20 ng/ml mouse macrophage colony stimulating factor (M-CSF) (BioLegend) or immediately after cell sorting or one day of culture (NPM1 cells). Cells were cultured with pHrodo Green *E. coli* BioParticles Conjugate for Phagocytosis (Thermo Fisher Scientific) for 2 hours. Cultured cells were washed, fixed with 4% paraformaldehyde, and stained with the CD11b antibody and DAPI. Sorted cells and NPM1 cells were stained with 5 μg/ml Hoechst 33342 without fixation. Stained cells were observed by fluorescence microscopy. Image analyses were performed using ImageJ.

### Histological analysis

For smear preparation, Cytospin (Thermo Fisher Scientific) was used and Diff-Quik (Sysmex Corporation, Kyoto, Japan) was used for staining. After ethanol dehydration and xylene permeation, specimens were sealed by TOLUENE SOLUTION (Fisher Scientific). Smear specimens were observed by optical microscopy to record morphological/structural characteristics.

### Establishment of the immortalised NMR cell line; NPM1

NMR PECs cultured with 10 ng/ml mouse M-CSF were immortalised by using a lentiviral vector encoding SV40ER. The vector and packaging plasmids pMD2.G and psPAX2 (Addgene, Watertown, MA, USA) were used to transfect X293T cells with polyethylenimine MAX transfection reagent (CosmoBio, Tokyo, Japan), according to the manufacturer’s instructions. The virus particles in the conditioned medium were collected and precipitated with polyethylene glycol. Precipitated viral particles were suspended in medium and added to NMR PECs cultured in 10 cm dish. Infected PECs were cultured at 32 °C in a 5% O_2_ and 5% CO_2_ atmosphere. The same medium for freshly isolated NMR cells was used and 20 ng/ml mM-CSF was supplemented. Medium was changed at 5–7 days. A month later, we stopped adding mM-CSF and found that cells still proliferated without cytokines. Cells ware passaged about 2 to 3 days. At each passage, the culture medium was discarded, and cells were washed by PBS. Then, 0.5 g/l Trypsin/0.53 mmol/l EDTA Solution (Nacalai Tesque) was added and incubated for 5 minutes at 32 °C. As for NPM1 cell line establishment, cell cloning wasn’t performed.

### LPS stimulation

NPM1 cells were stimulated by 1 μg/ml LPS (SIGMA-ALDRICH) for 24 hours, then the cells were used for further analysis.

### Induction of M1/M2-type macrophage in NPM1 cells

A modified version of the M1/M2 differentiation protocol was used in the current study based on a previous study^30^. NPM1 cells were cultured with 10 ng/ml mouse M-CSF for 6–7 days before differentiation. Next, the cells were stimulated toward M1, M2a, and M2c phenotypes by adding the following human/mouse cytokines and reagents: 10 μg/ml of LPS and 25 ng/ml of mouse (mM1) or human IFN-γ (hM1), 25 ng/ml mouse (mM2a) or human IL-4 (hM2a), 1 μM of dexamethasone (M2c), and saline (M0). Twenty-four hours after stimulation, the cells were harvested and gene expression was analysed.

### RNA isolation, cDNA synthesis and RT-PCR

Total RNA was extracted with PureLink RNA Mini Kit (Life Technologies) or TRISure (Nippon Genetics). cDNA was synthesized from total RNA by using ReverTra Ace qPCR RT Master Mix (Toyobo). mRNA expression levels were quantified with Step One Real-Time PCR System (Applied Biosystems) using a KAPA SYBR Fast qPCR kit (Nippon Genetics) with the following primers (all for NMR genes).

*Arg1* forward 5′-CATCGGAGCCCCTTTCTCAA-3′, reverse 5′-ACCAGCATATCTCAACGCCG-3′;

*Actb*: forward 5′-AGACCTTCAACACCCCAGCCATGT-3′,

reverse 5′-GGCCAGCCAGGTCCAGACGCAG-3′;

*CD11b*: forward 5′-AGAATGCAAGAGGCTTCGGG-3′, reverse 5′-CTGTGCTGTAGTCACACTGGT-3′;

*CD68*: forward 5′-TGTGAGGGTGCTCATACCCA-3′, reverse 5′-TGGCATTTCTGCAACTGAAGC-3′;

*CD69*: forward 5′-ATGTCCCACAACAGAGACCAG-3′, reverse 5′-GGACAGCACATGGGATAGGAA-3′;

*CD86* forward 5′-CTCGCTCCTCCCTGGCTAAT-3′, reverse 5′-TACCTGGTCCCATGGTGCAT-3′;

*CD163* forward 5′-AGTCCACTTGAGTCCCTTCACTA-3′, reverse 5′-TCCACTCTGCCACTACACTTG-3′;

*Csf1r*: forward 5′-TCAGACACACGGGGACCTAT-3′, reverse 5′-GGTGAAACCGTACCAGGGAG-3′;

*MHC class 2* forward 5′-CTGACCGAGTGAAGGAAGGG-3′, reverse 5′-AACAGACCTCCCTTGGGACT-3′;

*Ifnb*: forward 5′- ACAACGCAGCAGCAGTTT-3′, reverse 5′-GAGCAGCATTCTCCTTCC-3′;

*Ifng*: forward 5′-TGCGAGTCAAGATTTACAAAAGAA-3′, reverse 5′-TCTGGTTGCCTCCGGATTTT-3′;

*Il1b* forward 5′-GTGATGCACCCGTCCTAACA-3′, reverse 5′-GCCGGTTCAGATTCTCTCCG-3′;

*Il10* forward 5′-GTTGCCTGGTCTTACTGGCT-3′, reverse 5′-GCTTGATGTCTGGGTCGTGA-3′;

*Irf4* forward 5′-AGGGATTACGTCCCTGACCA-3′, reverse 5′-AGGTGGGGCACAAGCATAAA-3′;

*Irf5* forward 5′-CTTGGAAGCTGAGGACCTCTT-3′, reverse 5′-TCCACCCCCTCAGTGTACTT-3′;

*Itgax* forward 5′-GTATGAAGCCCACGACCCTG-3′, reverse 5′-ACCTGTCCATTTGCTTCCTCC-3′:

*Mpo* forward 5′-GCACAGGATACTTATGGGGCT-3, reverse 5′-CGAGGTCTCTACCTCCTCTGG-3′;

*Mrc1* forward 5′-AGCTTTGACTGCCTCGACTG-3′, reverse 5′-GTGGTCTTGTGTATTCACCTTTTGT-3′;

*Nos2* forward 5′-TCCCTTCCGAAGTTTCTGGC-3′, reverse 5′-GGAGTAGGCTGTGTGCACTT-3′;

*Siglec1* forward 5′-GGTCGCACATCCATGTCTCA-3′, reverse 5′-GTCCGGAGCACCTCATTTTC-3′;

*Tgfb* forward 5′-CCAGTGATACACCGGAGTGG-3′, reverse 5′-TTTGCTGTCACAGGAGCAGT-3′;

*Tlr3*: forward 5′-CCTTGTTGGGACTGTGGC-3′, reverse 5′-GGCAGGTGGCAATCTTCT-3′;

*Tlr4*: forward 5′-GCCTGCGTGAGACCTGAAAG-3′, reverse 5′-AGGGGATTAAAGCTCAGGTCC-3′;

*Tnfa*: forward 5′-ATGGCATGGATCTAACGG-3′, reverse 5′-CGGCTGACAGTATGGGTG-3′;

*Actb* was used for internal control. mRNA expression levels were calculated by ΔCT method.

### NMR gene and amino acid sequences

NMR gene and amino acid sequences were obtained the National Center for Biotechnology Information database and the Integrative Genomics of Ageing Group (Liverpool University). Amino acid sequence homology was assessed using CLC Sequence Viewer 8 (QIAGEN Bioinformatics, Redwood City, CA, USA) and Basic Local Alignment Search Tool (BLAST).

### Statistical analyses

Data were analysed with JMP Pro14 (SAS Institute Inc.) using Student’s *t*-tests. Values of p < 0.05 were considered statistically significant.

## Supplementary information

Supplementary figures

Supplementary figure legends
