# Supplementary material for: Flow cytometric identification and cell-line establishment of macrophages in naked mole-rats
Source: Sci Rep. 2019 Nov 29;9:17981. doi: 10.1038/s41598-019-54442-1 (PMC6884578; doi:10.1038/s41598-019-54442-1)
Supplement: Supplementary file 1 — Supplementary figures [file 41598_2019_54442_MOESM1_ESM.pdf]

Flow cytometric identification and cell-line establishment of macrophages in naked mole-rats

Haruka Wada[1,†], Yuhei Shibata[1,2,†], Yurika Abe[1], Ryo Otsuka[1], Nanami Eguchi[1], Yoshimi Kawamura[3,4], Kaori Oka[3,4], Muhammad Baghdadi[1], Tatsuya Atsumi[2], Kyoko Miura[3,4,5,*], and Ken-ichiro Seino[1*]

1 Division of Immunobiology, Institute for Genetic Medicine, Hokkaido University, Sapporo, Japan
2 Department of Rheumatology, Endocrinology and Nephrology, Graduate School of Medicine and Faculty of Medicine, Hokkaido University, Sapporo, Japan
3 Department of Aging and Longevity Research, Faculty of Life Sciences, Kumamoto University, Kumamoto, Japan
4 Biomedical Animal Research Laboratory, Institute for Genetic Medicine, Hokkaido University, Sapporo, Japan
5 Center for Metabolic Regulation of Healthy Aging, Kumamoto University, Kumamoto, Japan

† These authors contributed equally to this work

* Correspondence to;
Ken-ichiro Seino, M.D., Ph.D.
Division of Immunobiology, Institute for Genetic Medicine, Hokkaido University
Kita-15, Nishi-7, Kita-ku, Sapporo 060-0815 Japan
Tel: +81-11-706-5532 Fax: +81-11-706-7545
E-mail: seino@igm.hokudai.ac.jp

Kyoko Miura, Ph.D.
Department of Aging and Longevity Research, Faculty of Life Sciences, Kumamoto University
2-2-1 Honjo, Chuo-ku, Kumamoto 860-0811, Japan
Tel: +81-96-373-6852 Fax: Tel: +81-96-373-6852
E-mail: miurak@kumamoto-u.ac.jp

Fig. S1

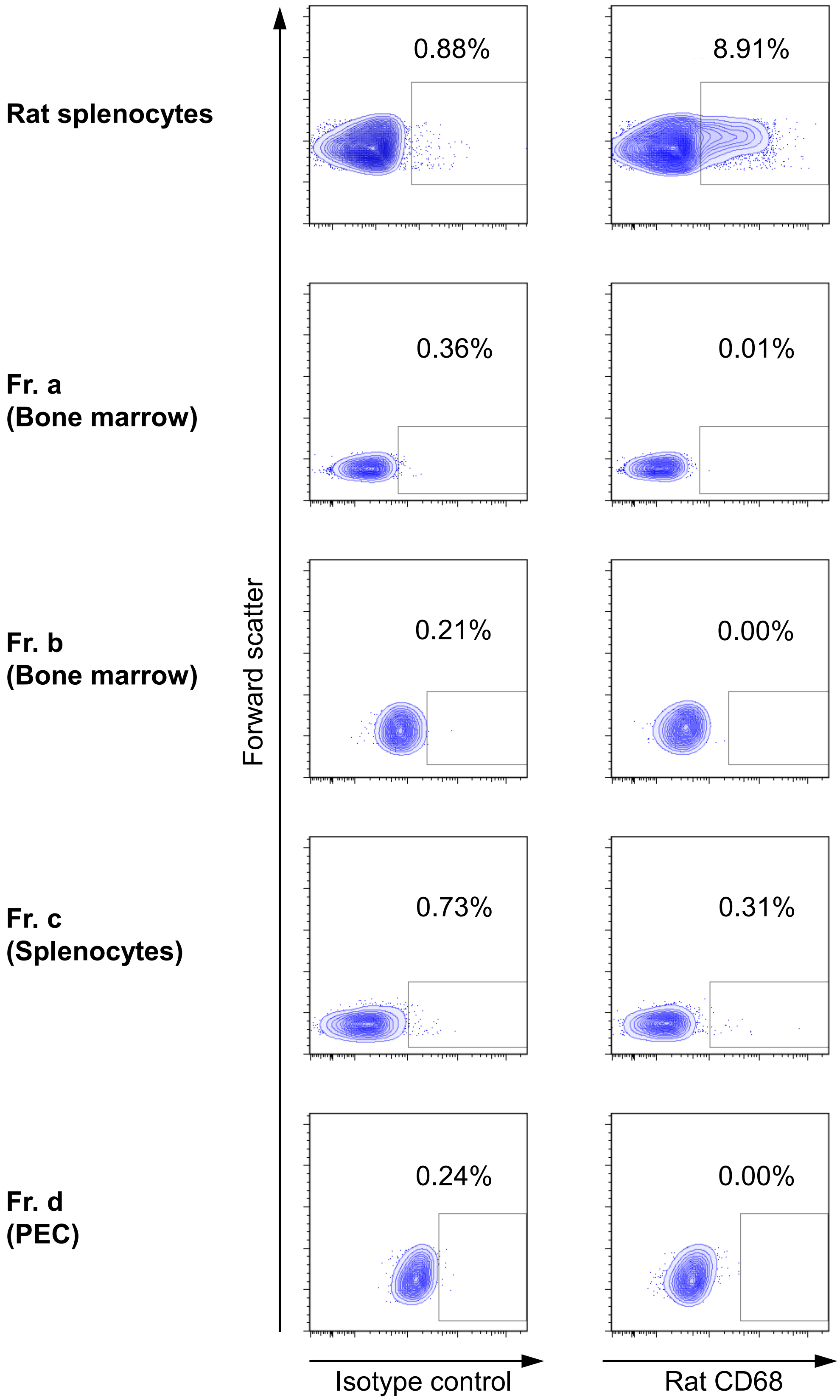
Rat splenocytes
Fr. a
(Bone marrow)
Fr. b
(Bone marrow)
Fr. c
(Splenocytes)
Fr. d
(PEC)
Forward scatter
0.88%
8.91%
0.36%
0.01%
0.21%
0.00%
0.73%
0.31%
0.24%
0.00%
Isotype control
Rat CD68

Fig. S2

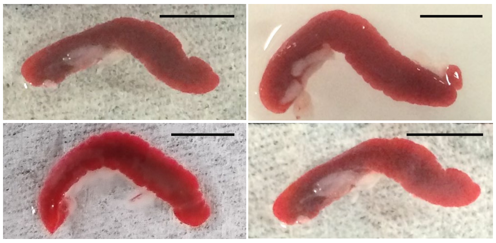

Fig. S3

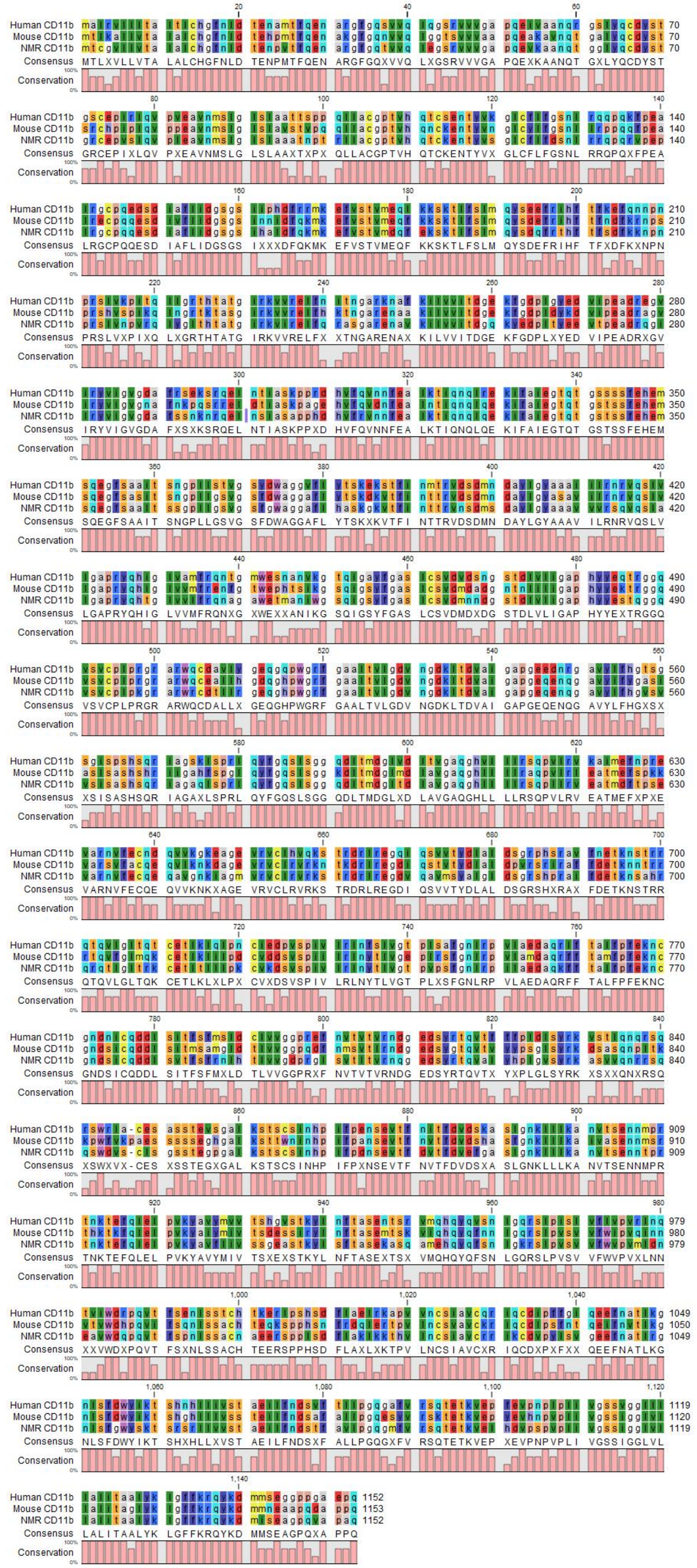

Fig. S4

a

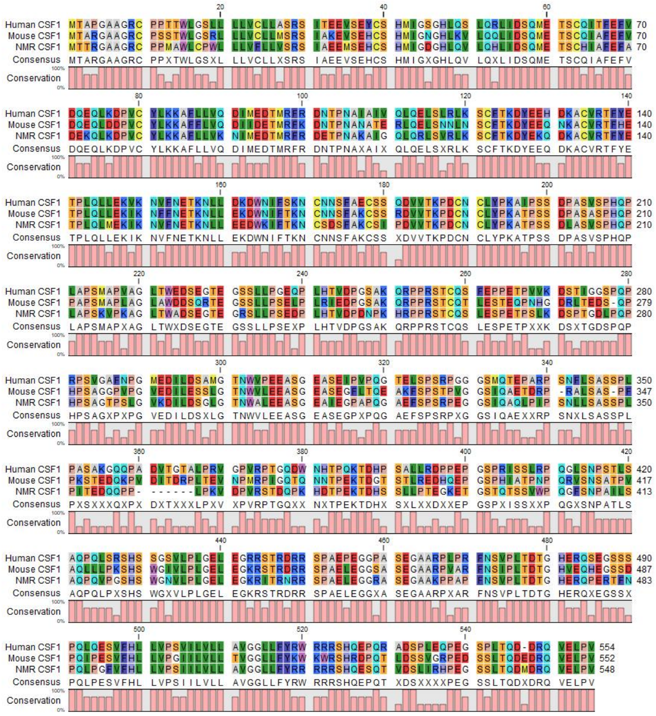
Human CSF1
Mouse CSF1
NMR CSF1
Consensus
Conservation

b

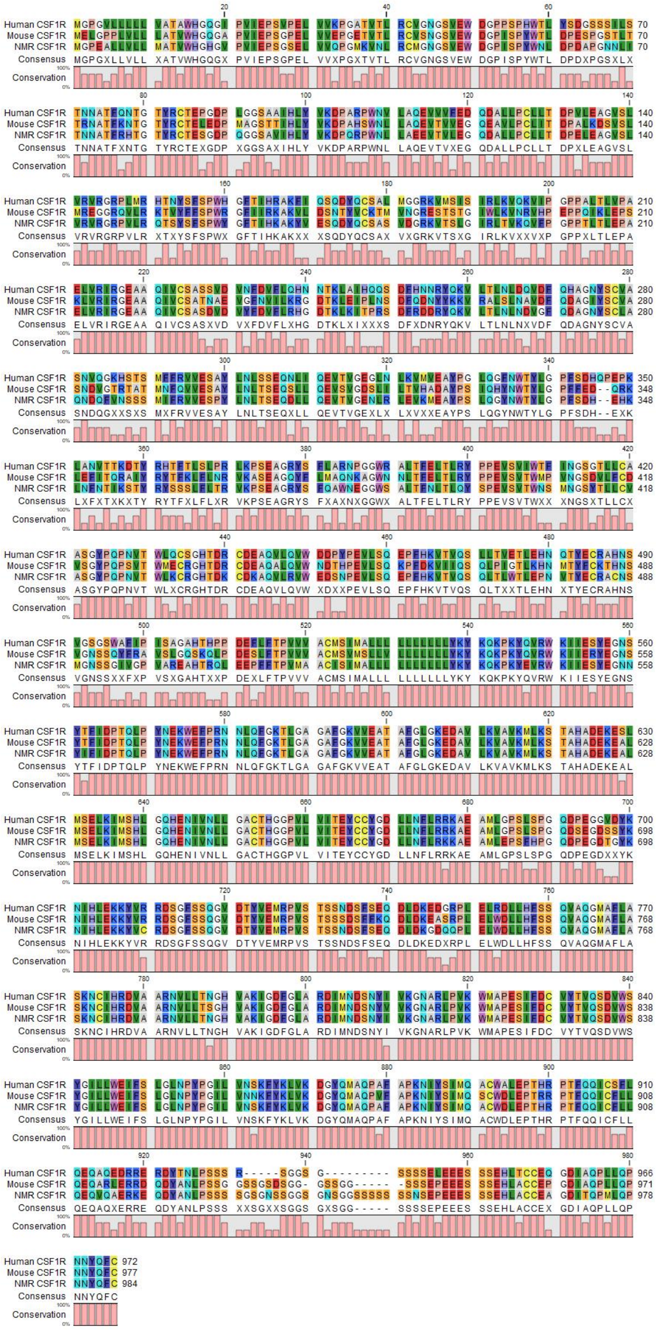
Human CSF1R
Mouse CSF1R
NMR CSF1R
Consensus
Conservation

Fig. S5

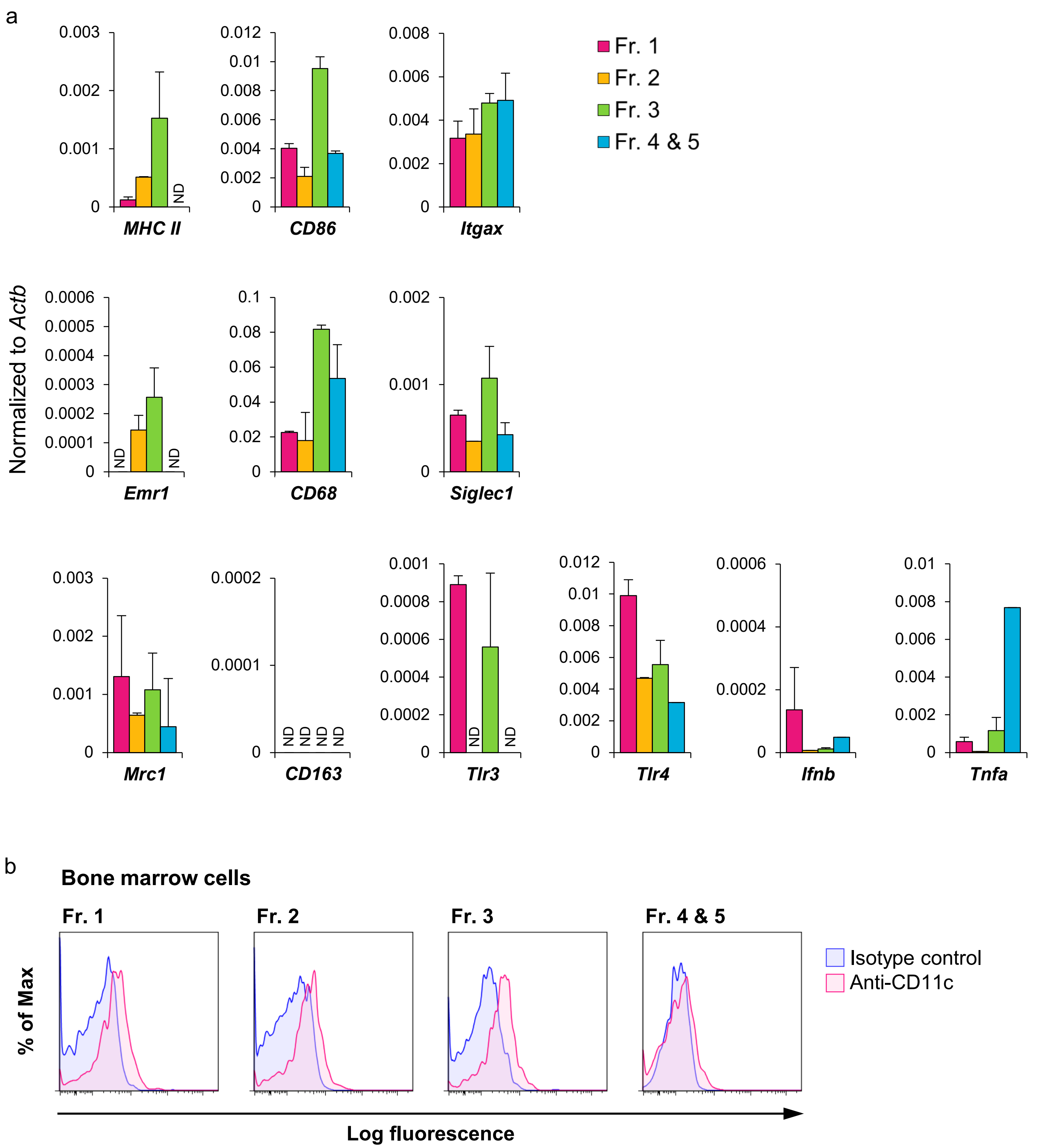

a
Normalized to Actb
MHC II
CD86
Itgax
Emr1
CD68
Siglec1
Mrc1
CD163
Tlr3
Tlr4
Ifnb
Tnfa
ND
Fr. 1
Fr. 2
Fr. 3
Fr. 4 & 5
b
Bone marrow cells
Fr. 1
Fr. 2
Fr. 3
Fr. 4 & 5
% of Max
Log fluorescence
Isotype control
Anti-CD11c

Fig. S6

a

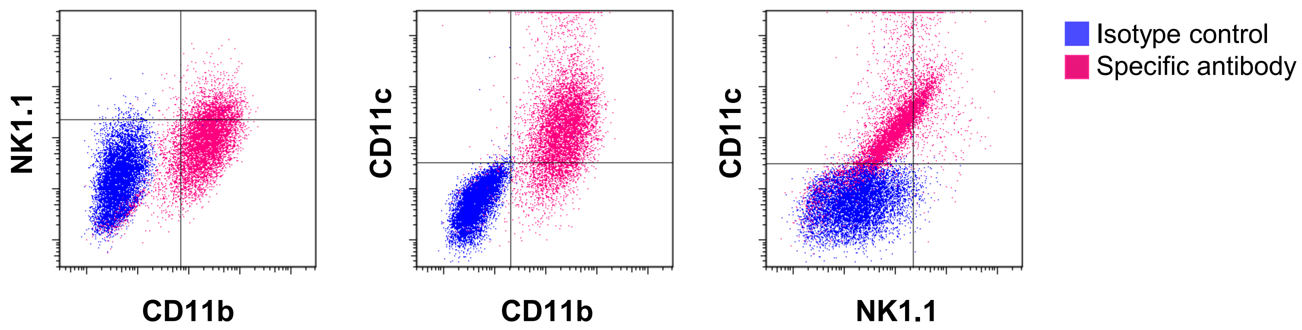
NK1.1
CD11b
CD11c
CD11b
CD11c
NK1.1
Isotype control
Specific antibody

b

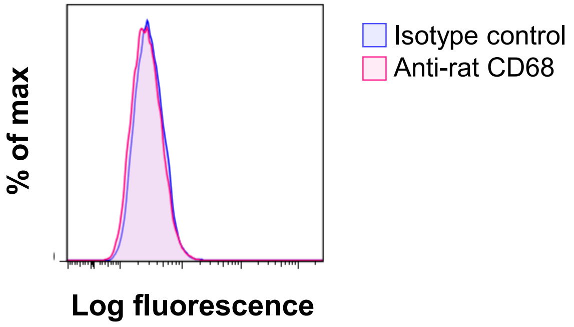
% of max
Log fluorescence
Isotype control
Anti-rat CD68
