# Supplementary material for: Flow cytometric identification and cell-line establishment of macrophages in naked mole-rats
Source: Sci Rep. 2019 Nov 29;9:17981. doi: 10.1038/s41598-019-54442-1 (PMC6884578; doi:10.1038/s41598-019-54442-1)
Supplement: Supplementary file 2 — Supplementary figure legends [file 41598_2019_54442_MOESM2_ESM.docx]

**Flow cytometric identification and cell-line establishment of macrophages in naked mole-rats**

Haruka Wada^1, †^, Yuhei Shibata^1,2, †^, Yurika Abe^1^, Ryo Otsuka^1^, Nanami Eguchi^1^, Yoshimi Kawamura^3,4^, Kaori Oka^3,4^, Muhammad Baghdadi^1^, Tatsuya Atsumi^2^, Kyoko Miura^3,4,5^*****, and Ken-ichiro Seino^1^*****

1 Division of Immunobiology, Institute for Genetic Medicine, Hokkaido University, Sapporo, Japan

2 Department of Rheumatology, Endocrinology and Nephrology, Graduate School of Medicine and Faculty of Medicine, Hokkaido University, Sapporo, Japan

3 Department of Aging and Longevity Research, Faculty of Life Sciences, Kumamoto University, Kumamoto, Japan

4 Biomedical Animal Research Laboratory, Institute for Genetic Medicine, Hokkaido University, Sapporo, Japan

5 Center for Metabolic Regulation of Healthy Aging, Kumamoto University, Kumamoto, Japan

^†^ These authors contributed equally to this work

*** Correspondence to;**

Ken-ichiro Seino, M.D., Ph.D.

Division of Immunobiology, Institute for Genetic Medicine, Hokkaido University

Kita-15, Nishi-7, Kita-ku, Sapporo 060-0815 Japan

Tel: +81-11-706-5532 Fax: +81-11-706-7545

E-mail: [seino@igm.hokudai.ac.jp](mailto:seino@igm.hokudai.ac.jp)

Kyoko Miura, Ph.D.

Department of Aging and Longevity Research, Faculty of Life Sciences, Kumamoto University

2-2-1 Honjo, Chuo-ku, Kumamoto 860-0811, Japan

Tel: +81-96-373-6852 Fax: Tel: +81-96-373-6852

E-mail: miurak@kumamoto-u.ac.jp

**Supplementary figure legends**

**Figure S1 Flow cytometric analysis of NMR cells using an anti-rat antibody**

Fleshly isolated rat splenocytes were hemolyzed and used for flow cytometry analysis. An anti-rat CD68 antibody and its isotype control were used for the analysis. A certain proportion of rat splenocytes were positively stained by the anti-rat CD68 antibody demonstrated that the practicality of the anti-rat CD68 antibody. Freshly isolated NMR bone marrow cells (BM), spleen cells (SP), and peritoneal cavity cells (PEC) were stained with the anti-rat CD68 antibody and its isotype control, and then analysed by flow cytometry. Cells in each fraction (Fr.) are shown. Numbers indicated % of positively stained cells in each specimen.

**Figure S2 Anatomical characteristics of NMR spleens**

Macroscopic images of NMR spleens. Sigmoidal shape and a slender form were observed. Scale bar: 1 cm

**Figure S3 Comparison of amino acid sequences of human, mouse, and NMR CD11b**

Amino acid sequences of CD11b in humans, mice, and NMR are shown. Homology of NMR amino acid sequences to mouse and human sequences was 72.77% and 75.35%.

**Figure S4 Comparison of amino acid sequences of M-CSF and its receptor CSF1R in humans, mice, and NMR**

(a) M-CSF (CSF1) amino acid sequences in humans, mice, and NMR are shown. Homologies of NMR amino acid sequences to mouse or human sequences were 67.21% or 71.71%. (b) CSF1R amino acid sequences of humans, mice, and NMR. Homologies of NMR amino acid sequences to mouse and human sequences were 74.09% and 79.31%.

**Figure S5 Gene expression analysis of NMR’s bone marrow cells**

(a) Gene expression of fractionated NMR bone marrow cells (Fig. 3B) were analysed. Gene expression levels are presented as means ± SD. ND: not detected. (b) Flow cytometry analysis of the fractionated NMR bone marrow cells using anti-mouse CD11c and its isotype control was performed.

**Figure S6 Flow cytometric analysis of NPM1**

NPM1 were analysed by flow cytometry. (a) Pink dots show specific antibody staining, and blue dots show each isotype control. (b) Magenta histogram shows anti-rat CD68 antibody staining, and blue histogram shows its isotype control.
